# Aortic Valve Stenosis: Diagnostic Approaches and Recommendations of the 2021 ESC/EACTS Guidelines for the Management of Valvular Heart Disease –A Review of the Literature

**DOI:** 10.26502/fccm.92920267

**Published:** 2022-06-27

**Authors:** Mukaram Rana

**Affiliations:** Department of Cardiology, Angiology, and Intensive Care Unit, Fulda Clinic, University Medicine Marburg-Campus Fulda, Germany

**Keywords:** Aortic Valve Stenosis, Aortic Stenosis, Diagnosis of Aortic Stenosis, Guidelines for the Management of Valvular Heart Disease

## Abstract

Aortic stenosis (AS) is the most common valvular heart disease in Europe and North America requiring a surgical or interventional treatment. Due to demographic changes with an aging population the burden of valvular heart diseases and especially the importance of aortic stenosis (AS) will be growing in future. As the onset of symptoms is associated with a decrease in life expectancy appropriate and early diagnosis are of utmost importance. However, insights of clinical practice underline diagnostic challenges which may lead to a delayed initiation of treatment with an adverse effect on the prognosis. The aim of this review is to display different diagnostic approaches that may be helpful in detecting patients with aortic valve stenosis. This review will focus on both non-invasive and invasive diagnostic approaches that can be implemented in clinical routine. Further-more, we will especially highlight recommendations of the 2021 European guidelines for the management of valvular heart disease.

**Methods:**

For this review a selective literature research on the databases PubMed and Google Scholar was conducted. Original articles, reviews and meta-analyses were included when meeting our search criteria. Following terms were used in different combinations: Aortic valve stenosis; Aortic stenosis; diagnosis of aortic stenosis; ESC Guidelines for the management of valvular heart disease.

## 1. Background

### 1.1 Epidemiology and etiology

Aortic stenosis (AS) is the leading primary valvular heart disease in Europe and North America and the second most common cardiovascular disease after arterial hypertension and coronary artery disease [1]. The prevalence increases with age varying from 0.2 % in the age group 50-59 years to 9.8 % among 80-89 years [2]. Congenital and acquired etiologies are known as driving factors in the development of AS. Degenerative calcific aortic stenosis remains the most common type of acquired AS [3]. Both the development of calcific AS and atherosclerosis show pivotal histopathological similarities in the initial phase of the disease [4, 5]. Endothelial damages caused by mechanical stress lead to lipid deposition and infiltration of the subendothelial tissue [6]. Infiltrating lipids undergo a process of oxidation which is promoted by reactive oxygen species (ROS). Consequently, they develop the capability of promoting further cytotoxic and pro-inflammatory effects [7, 8]. The uptake of oxidized low-density lipoproteins (LDL) by monocytes leads to the development of foam cells which play a key role in the development of atherosclerosis [9]. The production of pro-inflammatory cytokines accelerates further immunological cascades which subsequently result in fibrosing and calcifying processes leading to a limitation of cusp mobility [10]. Rheumatic aortic stenosis is the second common type of acquired AS and is considered the result of rheumatic fever occurring after an infection with group A streptococcus and leading to an immunological response with the production of autoantibodies [3, 11]. The mitral valve is affected more frequently than the aortic valve [12]. Most cases can be observed in children between 5 - 14 years [13, 14]. Advances in health care systems and the provided medical treatment in Western Europe and Northern America led to a noticeable decline in the incidence so that the disease is primarily prevalent in developing countries [15, 16].

### 1.2 Pathophysiology

In adults the aortic valve area (AVA) measures 3.0 to 4.0 cm^2^. A significant reduction in the aortic valve orifice area under 1.5 cm^2^ consequently leads to an increase of transvalvular pressure gradient with a subsequent decrease of blood flow from the left ventricle in the systemic circulation [17]. Furthermore, the pressure overload causes a compensatory left ventricle hypertrophy to maintain a sufficient cardiac output [18]. The reduced compliance resulting from the ventricle hypertrophy leads to an increased end-diastolic pressure contributing to the destruction of cardiomyocytes and thus facilitating a left ventricular dysfunction which may be associated with signs of heart failure [19]. Moreover, an impairment of the coronary flow reserve following from an inappropriate myocardial hypertrophy and an increased wall stress may lead to an imbalance of oxygen demand and oxygen supply causing the symptom of angina pectoris [20, 21].

### 1.3 Clinical features

An aortic valve stenosis can remain asymptomatic in the initial phase of the disease due to sufficient compensatory mechanisms. However, the 5-year risk of developing symptoms remains high [22, 23]. The onset of symptoms is associated with a poor outcome if there is no causal treatment [24, 25]. Cardinal symptoms of aortic stenosis are anginal chest pain, loss of consciousness, dyspnea, and signs of heart failure [26, 27].

### 1.4 Aim

Due to contemporary trends in demographic changes with an aging population diagnosis and therapy possibilities of AS will attract growing attention in the future. However, diagnostic uncertainties may lead to delayed diagnosis of AS which can be of prognostic relevance since the onset of symptoms is associated with a decrease in life expectancy. We therefore aimed to conduct a review of the contemporary literature and provide invasive and non-invasive diagnostic approaches on the basis of the latest European guidelines that may be beneficial for detecting aortic valve stenosis in daily clinical practice.

## 2. Diagnosis

### 2.1 Physical examination

Physical examination is an integral part of any primary doctor-patient contact and can play a key role for bedside diagnosis of suspected valvular heart disease. Auscultation of the heart as one element of the physical exanimation can be used to detect abnormal heart sounds and maybe helpful to decide whether further investigations are needed. Aortic valve stenosis can be characterized by a systolic murmur that is audible with its maximum intensity in the second right intercostal space. A radiation to the external carotid artery can be detected additionally [28–30]. In some cases, the high frequency component of the systolic murmur can be detected at the cardiac apex. This clinical sign is called Gallavardin phenomenon [31]. Although the stethoscope is one of the most useful diagnostic tools for clinicians conducting physical examinations it has its natural limitations. Auscultat-ion of heart sounds and the capability of distinguishing between different heart murmurs is highly experience-dependent which can lead to inconclusive findings among doctors with different levels of experience. Limited specificity and low sensitivity suggest the need of alternative diagnostic strategies [32]. In this context the current European guidelines do not recommend auscultation as a reliable diagnostic tool for the diagnosis of aortic valve stenosis.

### 2.2 Chest X-ray

Insights from the clinical routine affirm the observation that the chest x-ray is one of the initial imaging diagnostics in symptomatic patients with an undiagnosed aortic valve stenosis as these patients usually present with symptoms such as angina pectoris or dyspnea which need to be further investigated. The radiological findings are dependent on the stage of the disease and may be missing in the initial phase due to sufficient compensatory mechanisms. However, in an advanced stage of the disease cardiomegaly with an enlargement of the cardiac silhouette resulting from a left ventricle hypertrophy due to an increased afterload and an enlargement of the left atrium on the antero-posterior view may be detected. Furthermore, signs of heart failure, such as pulmonary venous congestion can be present. Although there are numerous signs that may indicate the presence of a relevant AS the chest X-ray remains a nonspecific tool in the diagnosis of AS and therefore plays a subordinate role. The current ESC guidelines therefore do not suggest the use of a chest X-ray as a sufficient diagnostic tool.

### 2.3 Electrocardiography (ECG)

In some cases, patients with AS may present electrocardiographic findings such as a left axis deviation as well as a positive Sokolow-Lyon index (S_v1_ + R_V5 or 6_ > 3.5 mV) as an expression of a left ventricular hypertrophy. Further optional signs such as left branch bundle blocks, T-wave negativations and ST depressions can also be present [28, 33, 34]. However, the ECG is not a sufficient diagnostic tool to detect or quantify any AS which highlights its subordinate role in the diagnosis of valvular heart diseases. Nevertheless, it must be considered that this limitation does not weaken the indication of an extensive use in the clinical routine since relevant comorbidities such as coronary heart diseases are usually present in these patient groups. As far as the current guidelines are concerned no proposal or explicit recommendations have been made by the authors.

### 2.4 Echocardiography

Echocardiography is the tool of choice for confirming the diagnosis of aortic valve stenosis. It is highly valuable for assessing the left ventricular ejection fraction, the valve morphology, the opening movement of the valve and the wall thickness. Furthermore, a semi-quantitative assessment of the extent of valve calcification can be performed as. The severity of aortic valve stenosis is quantified with the help of doppler measurement [17, 35, 36]. Three parameters are needed to classify the aortic valve stenosis: peak transvalvular velocity (Vmax), mean pressure gradient (Pmean) and aortic valve area (AVA). Low flow is defined by a stroke volume index ≤ 35 ml/m^2^. According to the current guidelines AS can be classified in four groups (Table 1) [37]:

#### 2.4.1 Peak transvalvular velocity (V_max_):

Peak transvalvular velocity (V_max_) is the systolic velocity across the aortic valve. It can be measured using continuous-wave (CW) Doppler ultrasound (CW doppler sonography) [38]. It must be considered that velocity measurements require an optimal patient and transducer positioning. The ultrasound beam needs to be aligned parallel to the aortic jet. The so-called velocity-time integral (VTI) and the mean pressure gradient (P_mean_) are measured by tracing the velocity curve [39]. It must be noted that at least three consecutive beats should be averaged in sinus rhythm and at least five consecutive beats with irregular rhythms, for example atrial fibrillation.

#### 2.4.2 Mean pressure gradient (P_mean_):

Mean pressure gradient (P_mean_) is another parameter which is needed to assess aortic stenosis severity. It represents the pressure gradient between the left ventricle and the aorta during the entire systole. The mean pressure gradient is calculated based on Bernoulli’s equation [40]. However, for clinical use the so-called simplified Bernoulli equation is used:

$$\text{Δ}p=4\;V^{2}$$

**Formula 1:** Simplified Bernoulli equation. Δp = mean pressure gradient (mmHg); V = velocity (m/s).

#### 2.4.3 Aortic valve area (AVA):

The calculation of the aortic valve area (AVA) is performed using the continuity equation. The continuity equation is based on the consideration that the stroke volume (SV) moving over the left ventricular outflow tract (LVOT) corresponds to the stroke volume (SV) passing through the aortic valve. Three parameters are needed to calculate the aortic valve area (AVA) [41]:

1. Diameter of the LVOT
2. Flow velocity in the LVOT (determined by pulsed-wave Doppler sonography)
3. Flow velocity across the aortic valve (detemination by continuous wave Doppler sonography)
$$AVA\times VTI_{AV}=CSA_{LVOT}\times VTI_{LVOT}$$
$$AVA=\frac{CSA_{LVOT}\times VTI_{LVOT}}{VTI_{AV}}$$

**Formula 2:** Continuity equation. AV = Aortic Valve; AVA= Aortic Valve Area (cm2); CSA= Cross Sectional Area (cm2); LVOT= Left ventricular outflow tract; VTI = Velocity Time Integral (m/s).

#### 2.4.4 Grading of Aortic stenosis (AS) severity

### 2.5 Cardial computer tomography (CCT)

The role of cardial computer tomography (CCT) for the diagnosis of AS and for the pre-interventional planning is crucial. CT imaging can provide information about the extent of valvular calcification which has been identified as a predictor for the progression and prognosis of aortic stenosis [42, 43]. Furthermore, aortic valve calcification scores measured by CCT can help assessing the severity of aortic stenosis which then would suggest further investigations [44]. According to the current ESC guidelines for the management of valvular heart diseases the measured calcification scores can be used to predict the probability for the presence of a severe aortic valve stenosis. A severe AS can be highly likely (men > 3000, women > 1600 Agatston units), likely (men > 2000, women > 1200 Agastson units) and unlikely (men < 1600, women < 800 Agatston units) [37]. Apart from this, CCT is used to for preinterventional planning. It provides information about vascular and valve calcification, aortic valve anatomy, annular size and shape, aortic root dimensions, distance of coronary ostia to the aortic valve plane which are necessary to avoid complications such as aortic root injuries or coronary occlusion [37, 45]. In this context CCT can be considered as an important diagnostic tool providing adjunctive information for the assessment of AS that is not only used in terms of uncertainties but also plays a crucial role for the preinterventional planning.

### 2.6 Dobutamine stress echocardiography (DSE)

Dobutamine stress echocardiography (DSE) is an additional diagnostic tool that should be considered according to the current guidelines whenever results remain inconclusive, especially in patients with low-flow low-gradient AS with reduced ejection fraction. Dobutamine stress echocardiography may help distinguishing between true severe AS and pseudo-severe AS in patients with [46]. This diagnostic approach assumes that AVA and P_mean_ are flow-dependent and the mismatch of a lower than expected transvalvular gradient is due to reduced transvalvular flow. Pharmalogical stress testing with dobutamine is used for further distinctions. The dobutamine infusion is usually started at 5 *μ*g/kg/min and can be titrated to maximum 20 *μ*g/kg/min. During titration P_mean_ and AVA should be measured regularly. True severe AS is diagnosed when AVA is ≤ 1 cm^2^ and P_mean_ ≥ 40 mmHg [47]. From this point of view DSE is highly valuable diagnostic tool which does not only provide information about the severity of AS but also about the contractile reserve which is of prognostic relevance [48].

### 2.7 Cardiac catheterization

In case of discrepant or inconclusive diagnostic findings right- and left-sided heart catheterization should be performed. Cardiac output is measured based on thermodilution or Fick equation using a Swan-Ganz catheter. Transvalvular gradient is obtained by simultaneous measurement of the pressure in the left ventricle and in the ascending aorta which can be performed with the help of a single dual-lumen catheter or with two separate catheters. Using the so called Gorlin equation the aortic valve area can be measured [49, 50].

$$AVA=\frac{\mathrm{Cardiac output}}{\mathrm{Heart rate X Systolic ejection period X}\;44,3\;X\sqrt{\text{Δ}P_{⬚}}}$$

**Formula 3:** Gorlin equation. Abbreviation: AVA = aortic valve area (cm^2^); ΔP = mean gradient (mmHg).

## 3. Conclusion

This review aimed to highlight the importance of various non-invasive and invasive diagnostic approaches for detecting AS in clinical practice. We could show that the current ESC 2021 guidelines for the management of valvular heart diseases consider echocardiography as the tool of choice for confirming the diagnosis of aortic valve stenosis. However, in cases of inconclusive results and measurements additional diagnostic approaches using cardial computer tomography (CCT), dobutamine stress echocardiography (DSE) and right- and left-sided heart catheterization should be considered. Auscultation, chest X-ray and ECG play a subordinate role due to their natural limitations and their low specificity and sensitivity for the diagnosis of AS.

## Figures and Tables

**Table 1:** Categories of aortic valve stenosis (AS).

High-gradient AS	Low-flow low-gradient AS with reduced ejection function	Low-flow low-gradient AS with preserved ejection fraction	Normal-flow low-gradient AS with preserved ejection fraction
AVA ≤ 1 cm^2^	AVA ≤ 1 cm^2^	AVA ≤ 1 cm^2^	AVA ≤ 1 cm^2^
P_mean_ ≥ 40 mmHg	P_mean_ < 40 mmHg	P_mean_ < 40 mmHg	P_mean_ < 40 mmHg
Peak velocity ≥ 4.0 m/s	SVi ≤ 35 ml/m^2^	SVi ≤ 35 ml/m^2^	SVi > 35 ml/m^2^
	LVEF < 50%	LVEF ≥ 50%	LVEF ≥ 50%

Abbreviations: AS = aortic valve stenosis; AVA = aortic valve area; LVEF = left ventricular ejection fraction; Pmean = mean pressure gradient; SVi= stroke volume index.

**Table 2:** Grading of Aortic stenosis [39].

	Mild AS	Moderate AS	Severe AS
**Peak velocity (m/s)**	2,6 - 2,9	3,0 - 4,0	≥4,0
**Mean gradient (mmHg)**	< 20	20 - 40	≥40
**Aortic valve area (AVA) (cm^2^)**	> 1,5	1,0 - 1,5	< 1,0
**Indexed AVA (cm^2^/m^2^)**	> 0,85	0,60 - 0,85	< 0,6
**Velocity ratio**	> 0,5	0,25 - 0,5	< 0,25
